# Predominance of Th2 polarization by Vitamin D through a STAT6-dependent mechanism

**DOI:** 10.1186/1742-2094-8-56

**Published:** 2011-05-24

**Authors:** Scott Sloka, Claudia Silva, Jianxiong Wang, V Wee Yong

**Affiliations:** 1 Hotchkiss Brain Institute and the Department of Clinical Neurosciences University of Calgary, Calgary, Alberta, Canada

## Abstract

**Background:**

Vitamin D has several reported immunomodulatory properties including the reduced generation of pro-inflammatory CD4+ T helper 1 (Th1) cells and the increase in levels of the anti-inflammatory Th2 subset. Less clear has been the impact of vitamin D on the pro-inflammatory Th17 subset, and whether and how vitamin D may preferentially drive the polarization of one of the T helper subsets.

**Methods:**

Using human peripheral blood-derived mononuclear cells and mouse splenocytes and lymph node cells in culture, we examined whether and how vitamin D preferentially skews T cells towards the Th1, Th2 or Th17 subsets. Mice afflicted with the multiple sclerosis-like condition, experimental autoimmune encephalomyelitis (EAE), were examined in vivo for the relevance of the tissue culture-derived results.

**Results:**

We report that the biologically active form of vitamin D, 1,25-dihydroxyvitamin D3 {1,25(OH)2D3}, consistently generates human and murine Th2 cells in culture, frequently leaving unchanged the levels of Th1/Th17 cytokines. As a result, the ratio of Th2 to Th1 and Th17 is increased by 1,25(OH)2D3. The upregulation of Th2 to Th1 or Th17 subsets by 1,25(OH)2D3 is enabled by an increase of the GATA-3 transcription factor, which itself is promoted upstream by an elevation of the STAT6 transcription factor. In mice, the alleviation of EAE severity by 1,25(OH)2D3 is accompanied by elevation of levels of GATA-3 and STAT6. Significantly, the efficacy of 1,25(OH)2D3 in ameliorating EAE is completely lost in mice genetically deficient for STAT6, which was accompanied by the inability of 1,25(OH)2D3 to raise GATA-3 in STAT6 null lymphocytes.

**Conclusions:**

These results of vitamin D promoting a Th2 shift through upstream GATA-3 and STAT6 transcription factors shed mechanistic understanding on the utility of vitamin D in MS.

## Background

Multiple sclerosis (MS) is an inflammatory and neurodegenerative disorder with widespread demyelination and axonal loss within the central nervous system (CNS). The underlying etiology remains undefined although both environmental and genetic factors play a role [1,2], resulting in the over-activation of various immune subsets that accumulate in the CNS to produce injury. Familial inheritance, cigarette smoking, viral infection, and ultraviolet (UV) light exposure may all contribute to the risk of MS [1,3].

Vitamin D deficiency has previously [4] and recently been suggested as another contributing factor in the pathogenesis of MS [5-7]. Several studies have reported an inverse association of sunlight exposure, available UV radiation and MS prevalence [5,8,9], implicating vitamin D since UV B radiation (280 to 315 nm) converts 7-dehydrocholesterol to previtamin D3 in the epidermal and dermal layers in humans; previtamin D3 is then converted by a thermal process to vitamin D3 [10]. Due to the changing angle of declination of the sun, vitamin D insufficiency is common in the winter months in latitudes north of 42 °N latitude [11]. Therefore, vitamin D is of interest as the biological correlate of available UV radiation, although it has been proposed that other factors could also be involved [12].

In humans, vitamin D3 undergoes hydroxylation in the liver to produce 25-hydroxyvitamin D3 {25(OH)D3}, the main circulating form of vitamin D. 25(OH)D3 can be further hydroxylated in the liver to 24,25-dihydroxyvitamin D3, or in the kidney to the immunologically active form of vitamin D, 1,25 dihydroxyvitamin D_3 _{1,25(OH)2D3} [10,13]. Many publications {reviewed in [13-15]} have reported extensively on the immunomodulatory properties of 1,25(OH)2D3. In particular, 1,25(OH)2D3 decreases T cell proliferation, increases the activity and frequency of regulatory T cells, alters the production of specific antibody isotypes, reduces activity of dendritic cells or makes them tolerogenic, and affects tissue-specific lymphocyte homing.

Naïve CD4-positive T helper (Th) cells can differentiate into either pro-inflammatory Th1 and Th17 subsets, or into Th2 subset with anti-inflammatory or regulatory activity [16,17]. Vitamin D has been found to elevate Th2 cytokines [18,19] and to reduce Th1 cytokine levels [20,21]; however, others have also found vitamin D to inhibit Th1 levels without affecting Th2 deviation [22], or to reduce EAE disease severity without altering Th1 or Th2 levels [23]. These results emphasize that there needs to be clarity on the activity and mechanism of vitamin D in CD4 Th1/Th2 differentiation. The literature on vitamin D and Th17 cells is still emerging, and vitamin D has been reported to reduce the level of Th17 cytokines in human studies [24], and to decrease Th17 cells in mice with colitis [25] or EAE [26].

Given the uncertain nature of the impact of vitamin D on T cell subsets, and the recent analysis in genetically altered or chimeric mice of the requirement of T cell expression of vitamin D receptors for amelioration of EAE [27], we have addressed the relationship between vitamin D and Th subsets, focusing on whether 1,25(OH)2D3 acts predominantly through altering one of the Th subsets, and of the attendant mechanisms. We first analysed human and mouse T cells in culture, and then extended to EAE studies. We elucidated a central role for STAT6 in regulating the vitamin D-polarization of Th2 cells to alleviate disease activity.

## Methods

### Isolation of T Cells

Human peripheral blood mononuclear cells (PBMCs) were isolated from the blood of healthy adult volunteers by Ficoll-Hypaque centrifugation [28]. The PBMCs were washed once with phosphate-buffered saline (PBS) and suspended in serum-free AIM-V medium (Invitrogen Life Technologies, Burlington, Ontario). To activate T cells in the PBMC populations, 96 well round-bottomed plates were coated with 10 or 1000 ng/mL of purified mouse anti-human CD3 (BD Pharmingen, Franklin Lakes, NJ) for a period of 3 h. From previous experiments (data not shown), the coating at 1000 ng/mL of anti-CD3 gives maximal activation of T cells measured by proliferation assays. Since the *in vivo *environment in humans is unlikely to lead to the maximal activation of T cells, a submaximal level of activation with 10 ng/ml anti-CD3 was also used in most experiments as both a comparison to maximal activation and to better reflect physiology. This submaximal level of activation may also permit the more sensitive measurement of experimental changes that affect T cell activation.

Human PBMCs were plated at a density of one million cells/mL of anti-CD3 coated 96 well plates (200 μ L/well, 200,000 cells per well). An additional 10 ng/mL of anti-CD28 (BD Pharmingen) was added as a suspension to all cultures, and cells were left for 3 days at 37°C in a 5% humidified CO_2 _incubator. In order to promote measurable levels of IL-17, some anti-CD3/CD28 activated cultures were further exposed to IL-23 (20 ng/mL) and IL-1β (10 ng/mL) [29] Specified sister cultures were further exposed to either 0.1, 1 or 10 nM of 1,25(OH)2D3 (BioMol, Plymouth Meeting, PA). In some experiments, certain PBMC preparations did not receive anti-CD3 or 1,25(OH)2D3, and the floating cells collected 3 days thereafter are referred to as unactivated T cells.

Flow cytometry analyses of the floating cells collected after 3 days of the initiation of anti-CD3 treatment indicated that CD3+ T cells constituted approximately 90% of the total cell population (data not shown). Of the CD3 cells, 60% were CD4+ and 40% were CD8+. For the remaining, approximately 8% were CD56+ natural killer cells, approximately 2% were CD19+ B lymphocytes, and less than 1% were CD14+ monocytes. There was no significant difference in the proportion of the various cell subsets between the unactivated, 10 and 1000 ng/mL anti-CD3 activated PBMC populations (data not shown). Since the majority of cells were T cells, henceforth this human culture population is referred to as T cells.

### Quantitative Real-Time polymerase chain reaction (qPCR)

For qPCR, T cell RNA was extracted using the RNeasy Mini Kit columns (Qiagen, Mississauga, ON). DNase treatment (M610A) was performed according to the manufacturer's instructions (Promega, Madison, WI). Total RNA extracted was reverse transcribed using Superscript II (Invitrogen). Resulting cDNA was subjected to real-time quantitative PCR using an iCycler (BioRad). Transcripts were quantified by real-time quantitative PCR on the iCycler using RT2 Real Time SYBR Green/Fluorescein PCR Master Mix (SA Biosciences, Frederick, MA). mRNA expression for each gene was calculated using a comparative cycle threshold method, and was normalized to the amount of the reference gene 18S rRNA (expressed as arbitrary units). All PCR primers were purchased from SA Biosciences (18S, PPH05666E; IFNγ, PPH00380B; IL-5, PPH00692A; IL-17, PPH00537B; TBX21/T-bet, PPH00396A/PPM03727A; GATA-3, PPH02143A/PPM05199A; RORC/RORγT, PPH05877A/PPM25095A; STAT6, PPH00760B; Notch 1, PPM04747A).

### ELISA

Cytokine production by human and mouse PBMCs was assessed after activation of cells for 72 h. Cytokines in culture supernatants were measured by ELISA according to the manufacturer's protocol. ELISA kits were purchased from Invitrogen. The IL-17 was of the IL-17F form. Data were analysed using a SpectraMax 384 (Molecular Devices Corporation, Sunnyvale, CA) according to the manufacturer's instructions.

### Disease induction in mice and EAE analysis

EAE was induced in female C57BL/6 mice (Jackson Laboratories, Bar Harbor, Maine), aged 8-9 weeks, by injecting subcutaneously (s.c.) 50 μg myelin oligodendrocyte glycoprotein (MOG)_35-55 _in Complete Freund's Adjuvant (CFA) (Fisher, Michigan USA) supplemented with 4 mg/ml of Mycobacterium tuberculosis on day 0 [30,31]. Intraperitoneal (i.p.) pertussis toxin (0.1 μg/200 μl, List Biological labs, Hornby, ON) was administered on days 0 and 2. 1,25(OH)2D3 (100 ng) was given every other day i.p. in 50 μL of DMSO, while 50 μL of DMSO was used as the vehicle control; this method of administering 1,25(OH)2D3 to mice has been reported by others [18,19,26,32,33]. Treatment was initiated at the time of MOG immunization. In addition to the wildtype mice, STAT6 -/- knockout (KO) mice on the C57BL/6 background (Jackson Laboratories, Bar Harbour, Maine) were utilized. Animals were assessed daily using a 15-point disease score scale [30,31] replacing the more commonly used 5-point scale since the 15-point scale differentiates individual limb disability, rather than grouping both fore- or hind-limbs together. This allows for a more sensitive characterization of disease progression. The 15-point scale is the sum of the disease state for the tail (scored from 0-2) and all 4 limbs (each limb is scored from 0-3). All animals were handled in accordance with the policies outlined by the Canadian Council for Animal Care and the University of Calgary.

### Statistical Analysis

Statistical analysis was performed using R version 2.8.1 (The R Foundation for Statistical Computing) and Matlab version 7.7 (The Mathworks, Natick, MA, USA). Statistical differences for cells in culture were addressed using ANOVA with Bonferroni correction for multiple comparisons. Statistical differences between groups of mice in the EAE experiments were evaluated using a nonparametric analysis Mann-Whitney *U *test. An alpha of 0.05 was selected for statistical significance.

## Results

### 1,25(OH)2D3 differentially shifts human T cells in favor of Th2

**Figure 1:**
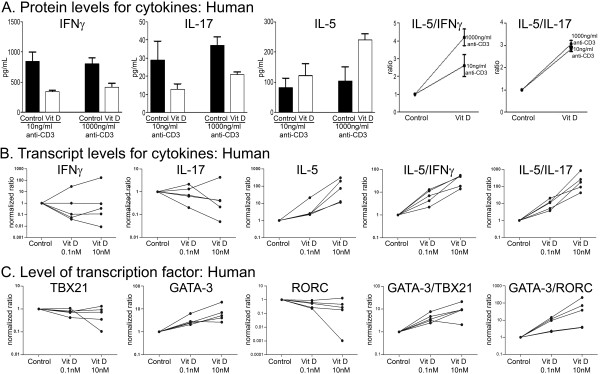
**Cytokine and transcription factor analyses show that 1,25(OH)2D3 polarizes human T cells in favor of Th2 rather than the Th1 and Th17 subsets**. A) ELISA results for T cells activated with two concentrations of anti-CD3 (10 and 1000 ng/mL) demonstrate that IFNγ and IL-17 concentrations decrease (p < 0.001), while IL-5 concentrations increase (p < 0.05), with the addition of 0.1 nM 1,25(OH)2D3 (denoted as Vit D in figure). Each bar is the mean ± SD of quadruplicate cultures and data displayed is from a single subject. The cytokine ratios were significantly increased between treated and untreated groups (p < 0.001). When analyzed across T cells from 4 subjects, we found that one subject did not alter IFNγ or IL-17 levels with 1,25(OH)2D3 treatment but IL-5 was elevated. Thus, across subjects, the increased ratio of IL-5/IFNγ and IL-5/IL-17 is a consistent outcome for 1,25(OH)2D3 treatment. B) qPCR results for 1000 ng/mL anti-CD3 activated T cells from five human donors (each line represents one donor) demonstrating that mRNA levels for both IFNγ and IL-17 were variably affected by 1,25(OH)2D3, but always increased for IL-5. In all five subjects, the ratio of IL-5 to both IFNγ and IL-17 was elevated by increasing concentrations of 1,25(OH)2D3. These results were reproduced in cells activated by 10 ng/mL anti-CD3 (data not shown). C) qPCR results of transcription factors for T cells from five donors activated with 1000ng/mL anti-CD3 show that while levels of TBX21 (which regulates Th1 polarization) and RORC (Th17) were largely unaltered by 1,25(OH)2D3, GATA-3 (which regulates Th2) transcripts were elevated, resulting in a consistent increase in the ratio of GATA-3 to both TBX21 and RORC levels. These results were reproduced in cells activated by 10 ng/mL anti-CD3 (data not shown).

We measured the changes in cytokine profile of T cells cultured with 1,25(OH)2D3. Supernatants from human T cells stimulated for 3 days with either 10 or 1000 ng/mL of anti-CD3, to reflect low and maximal activation of T cells, were analysed by ELISA for protein content of representative cytokines: interferon-γ (IFNγ) for Th1, interleukin (IL)-5 for Th2, and IL-17 for Th17 cells. Figure 1A shows that 1,25(OH)2D3 decreases IFNγ and IL-17 for both levels of anti-CD3 stimulation while increasing IL-5, indicating an elevation of Th2 to Th1/17 cells.

To examine the individual human response to 1,25(OH)2D3 more thoroughly, we next used qPCR of T cell samples cultured from several individuals for 3 days at 10 and 1000 ng/mL (1000ng/mL shown) of anti-CD3 as above. Figure 1B shows that the levels of IFNγ and IL-17 mRNA tended to be inconsistent and to change variably with 1,25(OH)2D3 treatment from one subject to the next; thus, levels of IFNγ and IL-17 mRNA may be increased, decreased, or unaltered for increasing concentrations of 1,25(OH)2D3. In contrast, IL-5 production consistently increased with 1,25(OH)2D3 exposure relative to no treatment, leading to a consistent elevation of the ratio of IL-5 to either IFNγ or IL-17 across T cell samples from the multiple subjects tested (Figure 1B). The mRNA results thus verified the polarization of CD4+ T cell subsets that was suggested by the ELISA assays of cytokines, with 1,25(OH)2D3 treatment consistently favoring Th2 anti-inflammatory over pro-inflammatory Th1 and Th17 subsets.

Specific transcription factors drive the formation of the Th subsets and the mRNA generated above for cytokine analyses were subjected to the levels of TBX21 for Th1, GATA-3 for Th2, and RORC for Th17 subsets. Similar to the above cytokine mRNA results, 1,25(OH)2D3 treatment reproducibly elevated the level of GATA-3 transcription factor mRNA across subjects, while producing variable responses for TBX21 and RORC mRNA levels amongst subjects (Figure 1C). When expressed as a ratio for both levels of anti-CD3 activation, 1,25(OH)2D3 produced an elevation of GATA-3 compared to TBX21 and RORC across all subjects analysed. Thus, the overall effect of 1,25(OH)2D3 on Th polarization could be attributed to its predominance of driving the Th2 subset.

### Murine cells respond similarly to human cells

**Figure 2:**
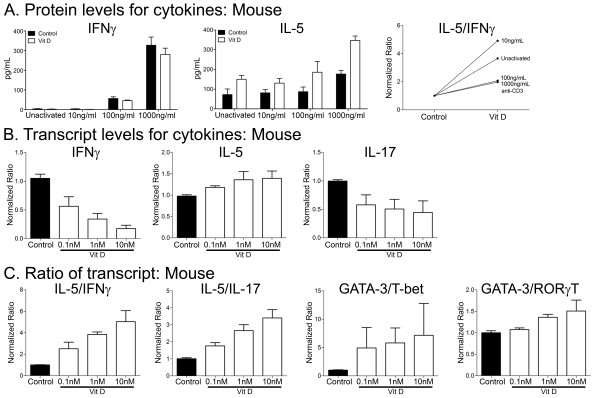
**Cytokines and transcription factors in mouse cells after 1,25(OH)2D3 treatment**. A) ELISA results for T cells activated with increasing concentrations of mouse anti-CD3 demonstrate increasing IFNγ and IL-5 concentrations, but 1,25(OH)2D3 suppresses the IFNγ concentration while increasing the IL-5 concentration. This results in an increase in the IL-5 to IFNγ ratio across all concentrations of anti-CD3. Each bar is the mean ± SD of quadruplicate cultures and data displayed is from a single mouse, repeated 3 times. B) qPCR results for 100ng/mL anti-CD3 demonstrate decreased IFNγ and IL-17 mRNA and increased IL-5 mRNA for increasing concentrations of 1,25(OH)2D3. C) The ratio of IL-5/IFNγ and IL-5/IL-17 mRNA rises with increasing concentrations of 1,25(OH)2D3. Similarly, the ratio of mRNA for GATA-3/T-bet and GATA-3/ROR-γT as determined by qPCR elevates with increasing concentrations of 1,25(OH)2D3.

We tested the response of murine T cells to 1,25(OH)2D3 in culture in order to allow subsequent transition to animal models. Mononuclear cells were isolated from mouse spleens and lymph nodes and were cultured for 3 days while being unactivated or stimulated with either 10, 100 or 1000 ng/mL of anti-CD3. We found that mouse IL-17 protein was difficult to detect reliably by ELISAs. As with the human ELISA results, murine T cells produce increasing concentrations of IL-5, but had reduced concentrations of IFNγ (Figure 2A), in response to 1,25(OH)2D3. The ratio of IL-5 to IFNγ protein was thus increased with 1,25(OH)2D3 treatment (Figure 2A).

RT-qPCR was next performed on murine T cells stimulated with 1000 ng/mL anti-CD3. IL-5 and GATA-3 transcripts were consistently elevated with increasing 1,25(OH)2D3 concentrations while IFNγ and IL-17 tended to be decreased. The ratios of IL-5 to IFNγ and IL-17 were thus upregulated with increasing concentrations of 1,25(OH)2D3, as were the ratios of GATA-3 to T-bet (the mouse equivalent of TBX21) and RORγT (the mouse equivalent of RORC) (Figure 2B), confirming a similar response in mouse and human cells.

### STAT6 but not Notch1 is elevated in human PBMCs with increasing 1,25(OH)2D3 concentrations

Since both IL-5 and GATA-3 were consistently elevated with increasing 1,25(OH)2D3 concentrations, we explored upstream factors that affect GATA-3 levels in human cells. Several factors have been reported to increase GATA-3 expression [34,35], including Notch1 and STAT6.

**Figure 3:**
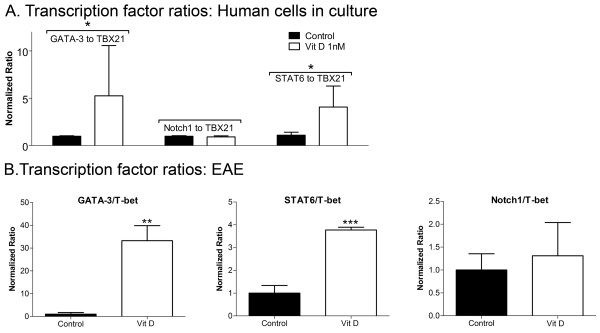
**Human PBMCs exposed to 1,25(OH)2D3 have increased GATA-3 to TBX21 ratios, associated with increased STAT6/TBX21 but not Notch1/TBX21 ratios**. A) Human PBMCs cultured for 3 days with 10 ng/mL of anti-CD3 demonstrate an increase in the STAT6 to TBX21 ratios by qPCR. N = 4 subjects, mean ± SD. B) Mouse PBMCs from EAE animals demonstrate an increase in GATA-3/TBX21 and STAT6/TBX21 ratios but not Notch1/TBX21 ratios by qPCR. N = 4 animals, mean ± SD, repeated twice. *P < 0.05; **p < 0.01; ***p < 0.001 compared to controls.

Human PBMCs stimulated with 10 ng/ml anti-CD3 were treated with 1nM of 1,25(OH)2D3 or left as untreated controls. After 3 days, RNA was harvested and analyzed using RT-qPCR. Figure 3A shows that the ratio of STAT6 to TBX21 transcripts increased with 1,25(OH)2D3 treatment, similar to the ratio of GATA-3 to TBX21, while the ratio of Notch1 to TBX21 remained constant. These results indicate that 1,25(OH)2D3 likely acts through STAT6 to increase GATA-3 levels, with no significant contribution through Notch1. Similar results are noted for cells activated with both 10 and 1000ng/mL of anti-CD3, and in the culture of murine PBMCs (data not shown).

### 1,25(OH)2D3 treatment of EAE-afflicted mice raises STAT6

We induced C57BL/6 wildtype mice for EAE and examined the amounts of GATA-3 and its potential regulators, STAT6 and Notch1, in the spleen and lymph nodes (Figure 3B) at peak clinical disease. To normalize across samples and for ease of comparisons, we expressed the results as a ratio to T-bet. We compared these data between EAE mice treated with 1,25(OH)2D3 or vehicle.

**Figure 4:**
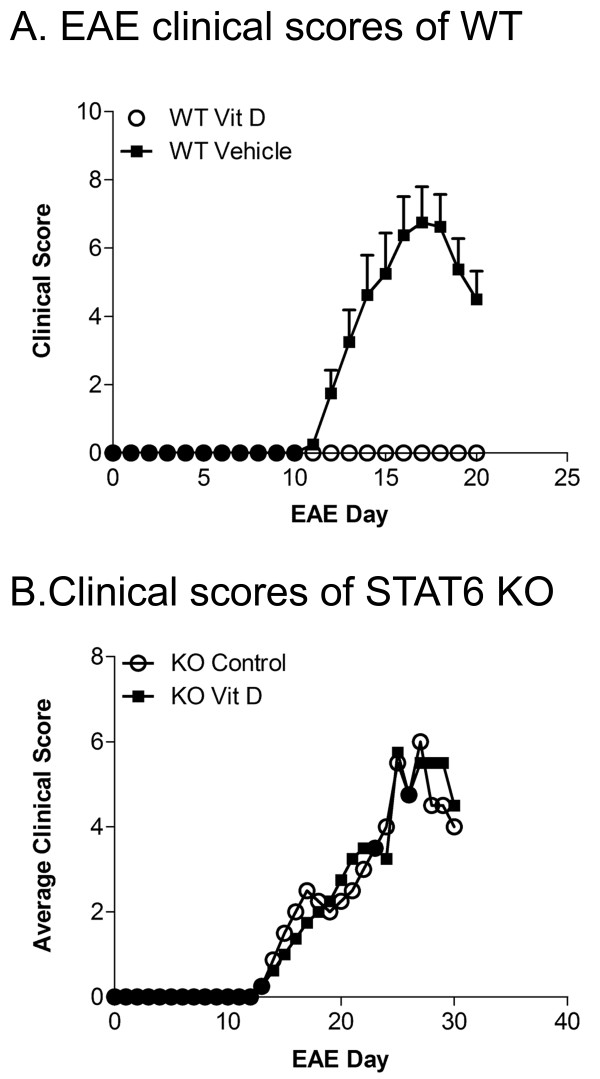
**Suppression of EAE clinical signs by 1,25(OH)2D3 occurs in wildtype but not STAT6 null mice**. A) Wildtype (WT) EAE-induced mice were treated with DMSO vehicle as controls, or with 1,25(OH)2D3 (denoted as Vit D). There was complete suppression of clinical signs in the 1,25(OH)2D3 treated group. N = 5 mice per group, and the trend of results has been reproduced in 2 other experiments. B) EAE was not suppressed in STAT6 null (knockout, KO) mice by treatment with 1,25(OH)2D3, using the identical dose regimen as for wildtype mice in panel A (100ng ip, beginning at MOG immunization, and administered every other day). N = 5 mice per group, repeated twice with similar results.

EAE has previously been shown to be largely abrogated with the treatment of 1,25(OH)2D3 [18,19,32,33,36,37]. We have reproduced this finding, and report here that the treatment every other day with 1,25(OH)2D3 essentially prevented C57BL/6 wildtype mice from succumbing to EAE signs (Figure 4A). At termination of the clinical scoring, spleens and lymph nodes were removed and RNA was isolated for qPCR. Figure 3B shows that relative to the vehicle treated EAE mice, the level of transcripts encoding GATA-3 to T-bet was high in 1,25(OH)2D3-treated wildtype mice. As well, levels of STAT6 but not NOTCH1 were elevated by 1,25(OH)2D3 treatment.

### STAT6 is required for the therapeutic effects of 1,25(OH)2D3 on EAE

Given that the upregulation of Th2-associated cytokine and transcription factor is paralleled by an increase of STAT6, we addressed whether STAT6 influences the therapeutic effect of 1,25(OH)2D3 in EAE. STAT6 knockout (KO) mice and wildtype controls were induced for EAE, and both succumbed to EAE. While there was a clear separation between the 1,25(OH)2D3-treated animals compared with the control animals in the wildtype groups (Figure 4A), the therapeutic effect of 1,25(OH)2D3 was lost when mice were without STAT6 (Figure 4B). These results emphasize that STAT6 is necessary for 1,25(OH)2D3 to alleviate EAE.

**Figure 5:**
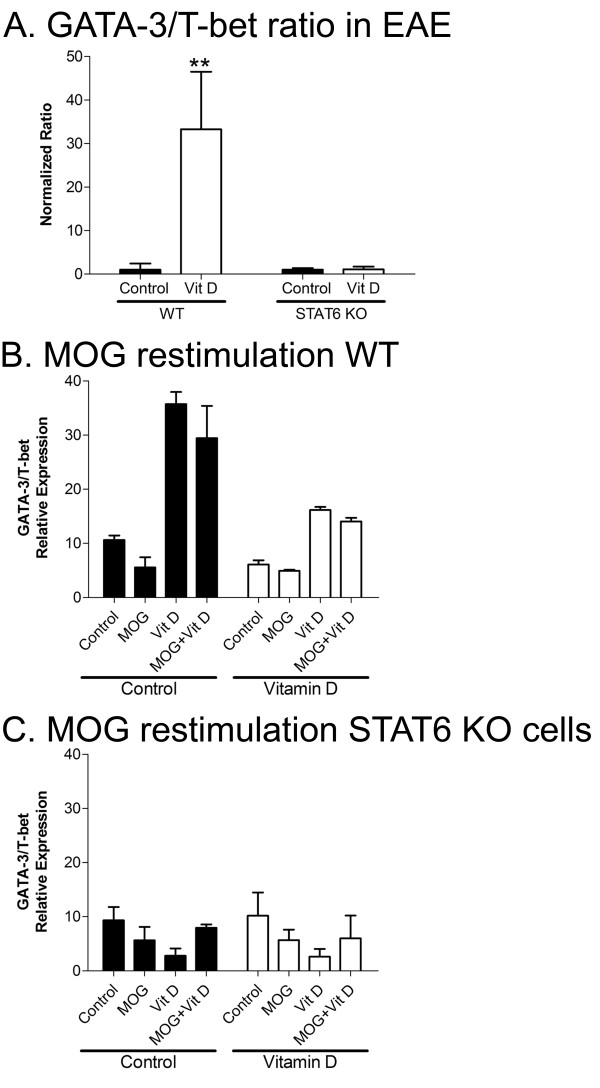
**The elevation of GATA-3/T-bet ratio in EAE-afflicted wildtype mice by 1,25(OH)2D3 did not occur in STAT6 null mice**. A) At sacrifice of the mice in Figure 4, spleens were removed for RNA whereby qPCR was performed. The GATA-3/T-bet ratios were increased by 1,25(OH)2D3 (denoted as Vit D) in wildtype (WT) mice but this elevation did not occur in STAT6 null (knockout, KO) mice. N = 5 per group, mean ± SEM, repeated twice. For panels B and C, spleen and lymph nodes from the animals sacrificed in Figure 4 were processed by Ficoll gradient centrifugation for lymphocytes and these were left untreated, or were treated ex vivo for 3 days with MOG (peptide 35-55), 1,25(OH)2D3 (denoted as Vit D), or MOG plus 1,25(OH)2D3. In wildtype mice (panel B) that were treated in vivo with vehicle or 1,25(OH)2D3, the ex vivo exposure to 1,25(OH)2D3, or MOG plus 1,25(OH)2D3, increased the GATA-3/T-bet ratio; importantly, this increase did not occur in STAT6 KO mice (panel C) whether they were exposed in vivo to vehicle or 1,25(OH)2D3. Bars are mean ± SEM, N = 5 per group, repeated twice.

Mice were killed at the conclusion of the EAE clinical scoring above, and their spleens and lymph nodes were extracted for RNA and analysed by RT-qPCR without further manipulation of tissue. While wildtype mice treated with 1,25(OH)2D3 clearly upregulated GATA-3 transcripts relative to T-bet, STAT6 KO mice did not have this response (Figure 5A), further substantiating the requirement of STAT6 for inducing an elevation of GATA-3 transcripts.

At sacrifice of the mice in Figure 4, lymphocytes were isolated and cultured for 3 days. Figure 5B shows that in wildtype cells from EAE mice previously treated in vivo with vehicle, the ex vivo treatment with 1,25(OH)2D3, with or without MOG restimulation, increased the GATA-3 to T-bet ratio. Significantly, the increase of GATA-3 to T-bet that occurred in wildtype mice did not occur in cells from the STAT6 KO mice (Figure 5C).

Overall, these results demonstrate that 1,25(OH)2D3 loses its therapeutic efficacy in EAE when STAT6 is absent, and this corresponds with the inability of STAT6 null cells to elevate GATA3 levels in response to 1,25(OH)2D3.

## Discussion

Evidence supporting the involvement of vitamin D in the risk of MS include an inverse correlation between MS prevalence and latitude that has been consistently observed [8,9,38], strongly suggesting a latitudinally-related environmental contribution to etiology. Available ultraviolet radiation, inversely correlated to latitude, is responsible for the peripheral conversion of 7-dehydrocholesterol to vitamin D3 in the epidermis. Therefore, vitamin D may be the biological correlate of available ultraviolet radiation conferring disease risk to a population. In MS patients, there is evidence of a seasonality of birth in MS patients (again suggestive of a seasonal environmental factor) [39], oral vitamin D intake appears to be protective [7] and vitamin D levels correlate inversely with disability [40]. In adult [41] or pediatric [42] MS populations, incremental increases in serum 25-hydroxyvitamin D3 levels are associated with reduced propensity for relapses. Two small trials have suggested that relapse rates may be reduced in patients taking oral vitamin D supplementation [43,44]. More recently, high dose intake (average of 10,000 IU/day) of vitamin D over 1 year in a study of 40 patients, appeared to reduce relapse rate in MS [45].

There is an increasing appreciation that Vitamin D exerts broad regulatory effects on cells of the adaptive and innate immune system. These include reducing antigen presentation through reducing the activity of dendritic cells or promoting their tolerogenic phenotype, affecting the polarization of monocytoid cells towards an M2 phenotype that produces anti-inflammatory cytokines (unpublished observations), altering B cell function, decreasing chemokine gradients and reducing tissue-specific homing [13-15,46]. A significant literature in humans also indicates that vitamin D increases the activity of regulatory T cells to prevent the excessive activation of autoreactive T cells [47,48]. These broad spectrum effects of vitamin D likely contribute to the apparent benefits of vitamin D in MS.

We sought in this manuscript to evaluate the impact of vitamin D on the polarization of CD4+ T helper subsets. We found a variable and inconsistent response of 1,25(OH)2D3 on generating human Th1 and Th17 subsets, and a predominance in elevating Th2 cells, resulting in the consistent outcome of increased Th2 to Th1 or Th17 ratios. These results were found by measurements of representative cytokines for each subset, and of their transcription factors. Similar results were found for mouse T cells in culture. Extending to studies in vivo, we found that mice treated with 1,25(OH)2D3 had significant generation of Th2 cells in the spleen and lymph nodes detected through GATA-3 upregulation, further emphasizing the predominance of Th2 cells generated through 1,25(OH)2D3 treatment.

In addressing the mechanism by which Th2 cells were generated, our results have highlighted the STAT6 transcription factor upstream of GATA-3, given the correspondence of elevation of STAT6 and GATA-3 in wildtype EAE mice treated with 1,25(OH)2D3, and of the significant loss of effect of 1,25(OH)2D3 in increasing GATA-3 levels in STAT6 null mice. Significantly, the deficiency of STAT6 by using null mice resulted in the inability of 1,25(OH)2D3 to alleviate EAE, and this is linked mechanistically to the failure in STAT6 null mice to elevate GATA-3 levels. Thus, our results have highlighted not only the predominance of generation of Th2 cells by vitamin D, but they have also revealed the intermediary role of 1,25(OH)2D3 in engaging STAT6 to produce Th2 polarization.

Vitamin D has also been reported to lose its efficacy in EAE when mice are deficient in IL-4 (20), IL-10 [49], Rag-1 [50], vitamin D receptor [51] and estrogen receptor signaling [50]. These results are consistent with our results of the lack of efficacy of 1,25(OH)2D3 in STAT6 null mice since IL-4 and IL-10 are cytokines produced by Th2 cells that require STAT6 for genesis, and the Rag-1 mutation leads to defect in the generation of T cells. A link between estrogen and vitamin D is demonstrated by the findings that estrogen regulates the level of the vitamin D receptor [50].

There are limitations to our current study that should be considered. The T cell population following 3 days of anti-CD3/CD28 stimulation of human peripheral blood-derived mononuclear cells constitutes approximately 90% purity, so it is possible that the effect of 1,25(OH)2D3 in generating Th2 predominance is indirect through cells that contaminate the T cell cultures. Moreover, our experiments utilize the known biologically active form of vitamin D, 1,25(OH)2D3, rather than the precursors vitamin D3 or 25-hydroxyvitamin D3. The last is the commonly measured form in humans due to its stability compared to 1,25(OH)2D3, so our results could be tempered by the potential differential rates in humans of converting 25-hydroxyvitamin D3 to 1,25(OH)2D3. As well, we did not determine whether other vitamin D metabolites, such as 24,25(OH)2D3, influence T cell function; different vitamin D metabolites can be found in MS patients and have been negatively correlated with outcomes of magnetic resonance imaging [52].

Another consideration is that while the concentrations of 1,25(OH)2D3 that we used in culture can be achieved in humans that are properly supplemented with vitamin D, it remains to be determined how much 1,25(OH)2D3 is found in mice injected in this study with intraperitoneal 1,25(OH)2D3. Our injection protocol and dose, however, reproduce those used by other groups [18,19,26,32,33]. Finally, it is of interest to determine whether 1,25(OH)2D3 would be equally efficacious in mice induced for EAE by active immunization with MOG, as in the current study, compared to mice elicited for EAE through the passive transfer of T cells; in these 2 context, different mechanisms are initially engaged to produce an inflammatory insult to the CNS, and their responses to vitamin D could help discern the key mechanisms for vitamin D in ameliorating EAE.

## Conclusions

The utility of vitamin D in multiple sclerosis is contributed by the polarization of helper T cells towards those that are of the regulatory, anti-inflammatory Th2 type, even when Th1 and Th17 levels are inconsistently modulated. The efficacy of 1,25(OH)2D3 in generating Th2 cells and in alleviating EAE requires the STAT6 transcription factor that lies upstream of GATA-3, raising the possibility that other approaches to stimulate STAT6 may increase the effectiveness of vitamin D. Thus, our results of the mechanistic understanding of vitamin D activity have relevance to the improvement of therapeutics to ameliorate MS.

## List of Abbreviations

1,25(OH)2D3: 1,25-dihydroxyvitamin D3; CFA: Complete Freund's Adjuvant; EAE: Experimental autoimmune encephalomyelitis; MS: multiple sclerosis; MOG: myelin oligodendrocyte glycoprotein; PBMCs: peripheral blood mononuclear cells; PBS: Phosphate buffered saline; Th: T helper cells.

## Competing interests

The authors declare that they have no competing interests.

## Authors' contributions

SS performed the majority of the experiments of this manuscript, and wrote the first draft of this manuscript. CS and JW provided technical support, and helped with deriving the results of Figures 1 and 2. VWY supervised this project, and edited and completed the writing of the manuscript. All authors have read and approved the final manuscript.
